# Roles Played by YY1 in Embryonic, Adult and Cancer Stem Cells

**DOI:** 10.1007/s12015-021-10151-9

**Published:** 2021-03-17

**Authors:** Gustavo Ulises Martinez-Ruiz, Abigail Morales-Sanchez, Angel Francisco Pacheco-Hernandez

**Affiliations:** 1 Research Division, School of Medicine, National Autonomous University of Mexico, Mexico City, Mexico; 2 Children’s Hospital of Mexico Federico Gomez, Mexico City, Mexico

**Keywords:** YY1, Cancer stem cells, Self‐renewal, Differentiation potential

## Abstract

Accumulating evidence strongly indicates that the presence of cancer stem cells (CSCs) leads to the emergence of worse clinical scenarios, such as chemo- and radiotherapy resistance, metastasis, and cancer recurrence. CSCs are a highly tumorigenic population characterized by self-renewal capacity and differentiation potential. Thus, CSCs establish a hierarchical intratumor organization that enables tumor adaptation to evade the immune response and resist anticancer therapy. YY1 functions as a transcription factor, RNA-binding protein, and 3D chromatin regulator. Thus, YY1 has multiple effects and regulates several molecular processes. Emerging evidence indicates that the development of lethal YY1-mediated cancer phenotypes is associated with the presence of or enrichment in cancer stem-like cells. Therefore, it is necessary to investigate whether and to what extent YY1 regulates the CSC phenotype. Since CSCs mirror the phenotypic behavior of stem cells, we initially describe the roles played by YY1 in embryonic and adult stem cells. Next, we scrutinize evidence supporting the contributions of YY1 in CSCs from a number of various cancer types. Finally, we identify new areas for further investigation into the YY1-CSCs axis, including the participation of YY1 in the CSC niche.

**Graphical Abstract:**

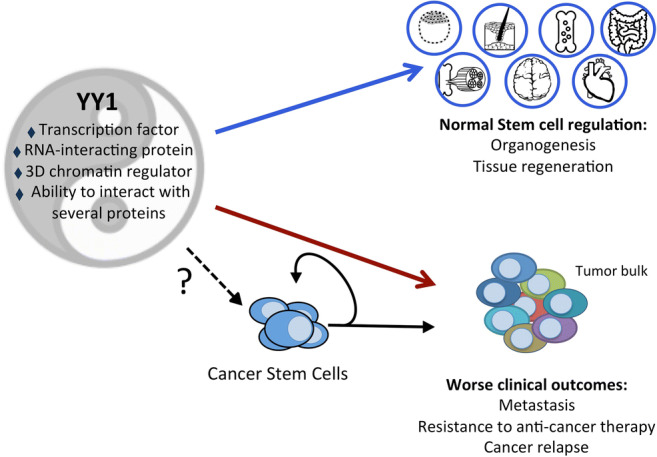

## Introduction

 Yin yang-1 (YY1) has been identified as a transcription factor with a dual function activating or repressing gene expression. It recognizes and binds to specific DNA sequences through its COOH-terminal region, which harbors four C_2_H_2_-type zinc finger motifs. YY1 also contains a REPO domain, which is an anchoring element 25 amino acids in length that recruits the polycomb group (PcG) proteins to DNA [1]. Thus, YY1 mediates a closed chromatin conformation by recruiting the polycomb repressive complex 2 (PRC2) containing histone methyltransferase EHZ2, which induces methylation of histone H3 at lysine 27 [2]. In contrast, YY1 mediates transcriptional activation by interacting with the INO80 chromatin remodeling complex in the regulatory regions of the genes [3]. Interestingly, the YY1-INO80 axis is also associated with homologous recombination-based DNA repair by potentially binding to a recombination intermediate structure [4]. Recently, it was shown that YY1 mediates the formation of genome-wide loops, contributing to the expression of cell type-specific genes [5–7]. Importantly, YY1 is able to interact with a number of RNA molecules, such as long noncoding RNAs (lncRNAs) [8–10], and proteins, including HDACs [11, 12], c-Myc [13], CTCF [14], p53 [15], Sp1 [16], and p300 [17]. Consequently, YY1 is implicated in several processes, such as transcriptional regulation, epigenetic modulation, chromatin remodeling, DNA repair, cell proliferation, cell death, differentiation, and development.

In cancer, the expression levels or functions of YY1 are commonly altered. Specifically, high expression levels of YY1 have been detected in several cancer types, including prostate [18, 19], breast [20], cervical [21, 22], glioma [23, 24], gastric [25], melanoma [26], liver [27], lung [28], etc. Notably, YY1 expression levels correlate with worse clinical outcomes, such as metastasis, invasion, resistance to anticancer therapy, and cancer recurrence [29–31]. Furthermore, YY1 is involved in multiple molecular mechanisms, enhancing all hallmarks of cancer [31]. Therefore, YY1 is conceptualized as an oncogenic protein fueling the emergence of lethal cancer phenotypes.

The cancer stem cell (CSC) model suggests the presence of two functional cell types in a tumor: CSCs and non-CSCs. The first type is a highly tumorigenic subpopulation with stem-like functional features: self-renewal capacity and differentiation potential. Similar to normal stem cells (SCs), CSCs proliferate by undergoing symmetrical cell divisions, securing their self-renewal and differentiation potentials [32, 33]. Non-CSCs are nontumorigenic heterogeneous cancer cells, hierarchically generated from CSCs through asymmetrical cell divisions [34, 35]. The frequency of CSCs is variable from 0.1 % or less up to 40 % [36–39], and non-CSCs certainly form the bulk of a tumor. Importantly, CSCs lead to the emergence of highly lethal cancer phenotypes associated with metastasis, chemo- and radiotherapy resistance, and cancer recurrence.

YY1 triggers the development of deadly cancer phenotypes with enriched presence of highly tumorigenic cells in the tumors, suggesting that YY1 may regulate the CSC phenotype. Here, we present evidence linking these two fields of cancer research that share a common denominator, to be implicated in the generation of worse clinical scenarios. CSCs mirror the stemness properties of self-renewal and differentiation potential; thus, we describe the role of YY1 in the biology of embryonic and adult SCs as a biological framework to show the role of YY1 in CSCs in various cancer types. Finally, we present possible new areas for future studies into the YY1-CSCs axis.

## YY1 Regulates Early Embryonic Development

Stemness potential plays an essential role during embryonic development. Thus, evaluation of the relevance of YY1 to the regulation of stemness potential requires assessment of its role during embryonic development. Donohoe and colleagues observed a very early lethal phenotype in YY1 knockout mice because the embryos died during the peri-implantation period at the blastocyst stage [40]. The blastocyst stage is characterized by the presence of ICM, blastocoelic cavity, and trophoblasts. Importantly, YY1 ablation or downregulation decreased the expression of the master regulators of embryogenesis, Oct4 and Sox2, in the inner cell mass (ICM, also known as the embryoblast) [41]. Interestingly, trophoblasts that form the outer layer of the blastocyst express high levels of YY1 [40]. Similarly, YY1 downregulation has been associated with recurrent miscarriage in patients [42]. Therefore, YY1 is essential for early embryonic development.

Embryonic stem cells (ESCs) are isolated from the ICM during the blastocyst stage of embryonic development. Notably, YY1 is required for stemness potential of these cells. Three gene expression subsignatures, known as the Core, PRC, and Myc modules, establish the regulatory circuitry of ESC responsible for self-renewal and pluripotency [43–45]. The transcription factors Oct4, Sox2, and Nanog mediate the core gene expression module by forming specialized autoregulatory and feedforward loops via interactions with their own and target promoter sequences [44, 45]. The PRC module is defined as those gene co-occupied and regulated by Suz12, Eed, Phc1, and Rnf2 [43]. The Myc module regulates more genes than the Core or PRC modules. Notably, the Myc module is overrepresented in cancer tissue samples [43]. Importantly, YY1 is apparently associated with the three modules of stemness regulation in ESCs. Specifically, YY1 mediates transcriptional activation of the Oct4 and Sox2 genes by forming long-range interactions by looping [46]. Moreover, YY1 can interact with members of the BAF complex, such as Smarc4, in the regulatory regions of transcriptionally activated pluripotent genes Nanog, Oct4, Myc, and others [47]. Thus, YY1 is associated with the Core module by transactivating its crucial transcription factors. Additionally, YY1 is associated with the PRC and Myc modules by cointeracting with a subset of the regulatory regions of certain genes. Interestingly, YY1 polarizes its binding from these regulatory regions to ones associated with the Core module upon interaction with Smac4 [47]. Furthermore, YY1 and transcription factors of the Myc module, including Myc itself, cobind to a subset of regulatory DNA sequences to drive their transcriptional activation, highlighting that YY1 may contribute to the regulatory circuitry of ESC [48]. In agreement with the role of YY1 in the three regulatory modules of ESC, YY1 downregulation decreases pluripotency and concomitantly induces ESC differentiation [47]. Interestingly, YY1 drives active gene expression in ESCs by interacting with the INO80 remodeling complex, whereas the YY1-PRC2 complex axis mediates gene silencing [48]. Therefore, YY1 regulates the transcriptional landscape of ESCs and is thus essential for the biology of ESCs.

After the blastocyst is implanted in the uterus, the primitive streak is formed marking the initiation of gastrulation, which results in the formation of a trilaminar embryonic disc from a bilaminar embryonic disc originating from the ICM. Significantly, YY1 ablation results in failed migration of primitive streak-derived cells into the interior of the embryo. This effect results from persistent E-cadherin expression that inhibits epithelial-mesenchymal transition (EMT) and generation of three germ cell layers [49]. Thus, YY1 is essential for the establishment of three germ layers: ectoderm, mesoderm, and endoderm.

## YY1 Modulates the Stemness Potential During Organogenesis

 YY1 plays a critical role in the early stages of embryonic development; thus, YY1 is expected to play a significant role in organogenesis by regulating the generation of lineage-committed stem and progenitor cells and modulating their stemness potential. Generation of conditional YY1 knockout mice demonstrated the role of YY1 during organogenesis (Table 1). The following sections present and discuss the information on the role of YY1 in the stemness potential that governs organ formation and residing adult stem cells. To have a better understanding of the available information, it will be presented taking as reference the three germ layers.

**Table 1:** Role of YY1 in organogenesis

Germ layer	Tissue or organ analyzed	Mouse models	Major findings	Ref.
Ectoderm	Brain	• Disruption of one allele in ES cells by homologous recombination before microinjection into C57BL/6 blastocysts generated heterozygous YY1 mice. • Conditional YY1 ablation in cerebral cortex was achieved by generating the Emx1-Cre;YY1^f/f^ mouse line. • The Emx1-Cre^ER(t2)^;YY1^f/f^ mouse line induced time-dependent conditional YY1 KO in the cerebral cortex. • p53 ablation in cerebral cortex lacking YY1 expression was achieved by engineering the Emx1-Cre;YY1^f/f^ Trp53^f/f^ mouse line. • YY1 ablation in early neuroepithelial cells in the mid-hindbrain region was achieved by generating the mouse line Cre-En1;YY1^f/f^. • Ablation of YY1 in premigratory neural crest stem cells (NCSCs) was achieve by generating the Wnt1-Cre;YY1^f/f^ transgenic mouse line.	• Heterozygous YY1 KO embryos showed brain defects, such as altered brain symmetries and exencephaly. ▪ YY1 ablation in the cerebral cortex decreased the forebrain size by inducing apoptosis-mediated cell death. Notably, p53 KO partially rescued the YY1 ablation phenotype in this brain region. ▪ YY1 regulated earlier stages of neural progenitor cell differentiation by gene expression associated with mitochondrial function and protein translation. ▪ YY1 ablation in the mid-hindbrain region provoked perinatal death due to dorsal midbrain hypoplasia and cerebellar agenesis. ▪ Neuroepithelial cells lacking YY1 expression inhibited cell cycle progression and activated apoptosis. ▪ Homozygous deletion of the YY1 gene in NCSCs induced craniofacial and midbrain defects.	[40, 50–52]
	Skin	• Time-dependent homozygous deletion of the YY1 gene in postmigratory neural crest was achieved by generating Sox10-Cre^ER(T2)^;YY1 ^f/f^ transgenic mice. • YY1 was time-dependently ablated in the adult melanocytic lineage by generating the Tyr-Cre^ER(T2)^;YY1^f/f^ mouse line.	• Ablation of the YY1 gene in early stages of neural crest development reduced the melanocyte population. Additionally, these embryos presented a reduction in the size of the dorsal root ganglia. • The survival and proliferation rates of adult melanocyte stem cells were compromised after YY1 ablation.	[50, 53].
Mesoderm	Hematopoietic system	• Time-dependent conditional YY1 ablation was achieved by generating the Mx1-Cre;YY1^f/f^ mouse line. • Competitive congenic bone marrow transplantations were performed into lethally irradiated mice (CD45.1^+^) by using a mixture of CD45.2^+^ bone marrow progenitors (Mx1-Cre or Mx1-Cre;YY1^f/f^) with CD45.1 competitor cells at the 1:1 or 1:9 ratios. • Bone marrow transplantations were achieved using Mx1-Cre;YY1 ^f/f^ bone marrow progenitor cells that overexpressed either YY1 or its variant lacking the REPO domain.	• YY1 ablation in bone marrow progenitors induced a pancytopenic phenotype. • LT-HSC, ST-HSC, multipotent progenitor cells, and myeloid linages were increased by ectopic expression of YY1. • Ectopic expression of YY1 inhibited B-cell population. • YY1 ablation exhausted HSC population by lowering c-kit singling to induce the loss of the quiescence state. • Ectopic expression of YY1 or YY1ΔREPO rescued from the YY1 ablation phenotype.	[54]
	Hearth	• Ablation of the YY1 gene in mesodermal precursor cells was achieved by generating the Msp1-Cre;YY1^f/f^ transgenic mouse line. • YY1 ablation in cardiac progenitor cells (CPCs) was achieved by generating the Nkx2.5-Cre;YY1^f/f^ mouse line. • Y1 expression was knocked out in cardiomyocytes by generating the α-MHC-Cre;YY1 ^f/f^ mouse line.	• Specific deletion of the YY1 gene in mesodermal cells resulted in early embryonic lethality due to the loss of the CPC population. • Conditional ablation of YY1 in the CPCs during cardiac development resulted in developmental defects. • Deletion of YY1 in cardiomyocytes provoked congenital defects in the heart.	[55, 56].
	Skeletal muscle	• YY1 ablation in muscle stem cells was achieved by generating the Pax7-Cre;YY1^f/f^ mouse line. • Time-dependent YY1 ablation in satellite cells was achieved by generating the Pax7-Cre^ER(T2)/+^;YY1^f/f^ mouse line. • Conditional YY ablation in skeletal muscle cell was achieved by generating the Myo-Cre;YY1 ^f/f^ mouse line	▪ Ablation of the YY1 gene in muscle progenitor cells induced neonatal death by suffocation due to insufficient diaphragm development. ▪ Injury-induced muscle regeneration was not correctly performed by adult satellite cells lacking YY1 expression. ▪ Loss of YY1 expression in satellite cells induced transcriptional activation of mitochondrial genes that are normally repressed by the YY1-PCR2 complex. ▪ Glycolytic HIF1α-responsive genes were inhibited in muscle stem cells lacking YY1 expression due to the absence of YY1-mediated stabilization of HIF1-α.	[57, 58]
Endoderm	Intestine	• Time-dependent YY1 ablation in intestinal epithelium was achieved by generating the Villin-Cre^ER(T2)^;YY1^f/f^ mouse line. • Intestinal Lrg5^+^ stem cells lacking YY1 expression were traced by crossing Villin-Cre^ER(T2)^;YY1 ^f/f^ mice with Lrg5^+^-EGFP-Ires-Cre^ER(T2)^ mice. • YY1 ablation in the intestinal epithelium was achieved by generating the Shh-Cre;YY1^f/f^ mouse line.	▪ Loss of YY1 expression in the intestinal epithelium was incompatible with life. ▪ Intestinal Lgr5^+^ stem cells lacking YY1 expression differentiated and migrated to the villi leaving their crypt base localization. ▪ YY1 was essential for the maintenance of the stemness potential of intestinal stem cells by regulating the gene expression associated with the mitochondrial function.	[59, 60].
	Lung	• YY1 ablation in the lung mesenchyme was achieved by generating Dermo-Cre;YY1 ^f/f^ or Shh-Cre;YY1^f/f^ mice	• The loss of YY1 expression in the lung mesenchyme compromised the lung development. Importantly, club cells and type-1 pneumocytes were dramatically decreased in the pulmonary epithelium. • Deletion of the YY1 gene in the pulmonary epithelium resulted in respiratory failures at birth. Activation of apoptosis and reduced proliferation were detected in the pulmonary epithelium lacking YY1 expression. Additionally, several cell types were lost due to YY1 ablation, such as club, ciliated, goblet, and smooth muscle cells.	[61, 62]

## YY1 Regulates Stem Cells Derived from the Ectodermal Lineage

The role of YY1 in stem cells associated with the brain and epidermal development from the ectoderm germ cell lineages was investigated using conditional KO mice (Table 1).

### Role of YY1 in Brain Development

YY1-mediated functions have been extensively characterized in the brain. The earliest description of the effects of YY1 on embryonic development was reported by Donohae et al. The authors demonstrated that a small proportion of embryos carrying heterozygous YY1 allele ablation exhibited growth and neurulation defects and structural brain alterations [40]. Conditional knockout of YY1 in premigratory neural crest cells was shown to induce embryonic death at E14.5, presenting midbrain and craniofacial defects. Additionally, a marked reduction in neural crest derivatives located in the enteric nervous system, dorsal root ganglia, and skin was observed in this model [50]. Dong and colleagues demonstrated that Cre-mediated deletion of the YY1 gene in the mid-hindbrain region induced hypoplasia and cerebellar agenesis by inhibiting cell cycle progression and inducing apoptotic death in neuroepithelial stem cells in a p53-dependent manner [51]. Another study demonstrated that knockout of the YY1 gene during cortex development decreased the forebrain size due to reduced proliferation and increased apoptotic death in cortical progenitor cells [52]. Interestingly, the ablation of YY1 affected neuronal progenitor cells (NPCs) at the earlier stages of differentiation during cortex development [52]. Similarly, Beagan et al. showed that YY1 is essential for NPC commitment by mediating the formation of specific DNA loops that attract distal and proximal regulatory elements to genomic regions demarcated by CTCF [6]. Thus, loss of YY1 compromises stemness potential of neural crest stem cells and progenitors cells, such as NPCs.

Determination of the molecular mechanisms governed by YY1 in NPCs required high-throughput technologies. Specifically, RNA-seq, YY1 ChIP-seq, and functional analyses were performed in NPCs after YY1 ablation. The lack of YY1 expression downregulated direct target genes associated with mitochondrial metabolism and protein translation [52]. On the other hand, a decrease in the cortex size induced by YY1 ablation was partially rescued by inhibiting cell death by knockout of the p53 gene [52]. YY1 negatively regulates p53 function [15, 63], and this molecular mechanism appears to be essential for brain development. Similarly, YY1 deletion in the mid-hindbrain region induced p53-dependent cell death [51]. Interestingly, YY1 knockout in this region compromised YY1-mediated Wnt1 transcriptional activation [51]. Importantly, ectopic expression demonstrated that Wnt1 is a key factor that regulates mid-hindbrain size [64]. Overall, studies using all these mouse models demonstrated that YY1 modulates brain development by regulating the proliferation, cell death, mitochondrial metabolism, and 3D chromatin configuration. Importantly, alterations in the human YY1 gene lead to craniofacial abnormalities accompanied by deficient intellectual abilities such as those observed in YY1 and Gabriele-de Vries syndromes [65, 66]. Investigations of patients diagnosed with YY1 syndrome showed that the affinity of YY1 interaction with its target DNA sites is reduced, provoking the global loss of H3K27 acetylation [65]. Thus, YY1 is a key regulator of cerebral organogenesis, and alterations in YY1 lead to several brain pathologies.

### Role of YY1 in Skin Development

Neural crest (NC) cells are an embryonic subpopulation involved in the generation of diverse cell types and tissues, such as connective tissue, cartilage, bone, and endocrine and melanocyte cells. Knockout of the YY1 gene in premigratory NC cells lead to embryonic lethality at E14.5. Additionally, ablation of YY1 reduced NC derivatives, including melanocytes, at E13.5 [50]. Moreover, time-dependent conditional ablation of YY1 in postmigratory NC cells at E10.5 decreased the melanocyte subpopulation [50]. Cre-mediated YY1 ablation in the melanocytic lineage was not lethal; however, these mice exhibited lighter skin pigmentation and hair graying [50, 53]. Thus, YY1 is essential for the stemness potential of both embryonic and adult melanocyte stem cells.

## YY1 Regulates Stem Cells Derived from the Mesoderm Lineage

The mesoderm is the last germ layer formed in the trilaminar embryonic disc and marks the beginning of the gastrulation stage of embryonic development. Experiments using YY1 KO embryos showed that YY1 plays an essential role in the formation of the mesoderm by regulating primitive streak formation that precedes the gastrulation stage [49]. Analysis of the organs and tissues generated from this layer demonstrated the regulatory role of YY1 in heart, muscle, and hematopoietic development.

### YY1 Regulates the Hearth Development

The Mesp1 gene encodes an essential transcription factor expressed in all cardiac mesodermal cells [67]. A conditional YY1 KO mouse line with ablation of the YY1 floxed alleles under Mesp1-Cre expression was engineered to investigate the role of YY1 in early embryonic heart development. YY1 ablation in cardiac mesodermal cells resulted in embryonic lethality due to a decrease in the cardiac progenitor cell (CPC) population [55]. Additionally, conditional YY1 KO in CPCs induced embryonic lethality, which was attributed to cell cycle inhibition, impaired EMT, and downregulation of several direct YY1 targets [56]. Concordantly, ectopic YY1 overexpression in ESC-derived CPCs maintained their limited stemness potential by regulating the epigenetic state of CPC-associated genes [68]. Importantly, overexpression of YY1 in embryonic stem cells enhanced the generation of CPCs [55]. Additionally, ablation of YY1 in cardiomyocytes provoked congenital abnormalities in cardiac histology due to decreased proliferative potential and enhanced activation of apoptosis in cardiomyocytes [56]. Overall, YY1 regulates stemness potential needed for the generation of CPCs, maintains limited stemness potential of CPCs, and is required for proliferative potential of cardiac lineage-committed cells to generate cardiomyocytes.

### YY1 Regulates the Muscle Development

Embryonic muscle progenitor cells express the transcription factors Pax3 and Pax7 (paired box proteins 3 and 7) necessary for the formation of fetal myofibers and skeletal muscle [69, 70]. YY1 was knocked out in Pax7^+^ cells to determine its role in muscle progenitor cells [57]. The data indicated that knockout mice died by suffocation after birth due to insufficient development of the diaphragm muscle evidenced by reduced levels of MyHC and troponin T proteins and concomitant loss of muscle fibers. Additionally, YY1-ablated mice had a smaller body size than that of the control animals [57]. Interestingly, ablation of YY1 in skeletal muscle cells during embryonic development did not induce significant changes at birth; however, the animals exhibited postnatal growth delay, showing a dwarf-like phenotype [58]. Additionally, these mice were characterized by exercise intolerance due to downregulation of YY1-mediated mitochondrial bioenergetics after YY1 ablation. Therefore, YY1 plays an essential role in muscle development.

### YY1 Regulates Adult Muscle Stem Cells

In addition to the role of YY1 in organogenesis of skeletal muscle, YY1 also participates in muscle regeneration by regulating the metabolism of satellite cells. Since YY1 ablation in embryonic muscle stem cells is deleterious, a conditional YY1 KO mouse line was engineered for inducible YY1 ablation in Pax7^+^ satellite cells [57]. This mouse model did not show any abnormalities up to 1 year after YY1 ablation; however, injury-induced muscle regeneration in the animals resulted in a small fiber size with severe atrophy [57]. Similarly, the embryos harboring skeletal muscle cells lacking YY1 expression did not have detectable abnormalities [58]. Importantly, activation of YY1-ablated Pax7^+^ cells by injury resulted in low proliferative potential, which was rescued by lentivirus-mediated ectopic expression of YY1 [57], highlighting the role of YY1 in the expansion of activated muscle stem cells. RNA-seq and YY1 ChIP-seq assays were used to identify molecular pathways regulated by YY1, and the loss of YY1 inhibited cell cycle progression and reactivated silenced target genes of YY1 associated with mitochondrial function [57]. Interestingly, YY1 ablation in differentiated skeletal muscle cells compromised the mitochondrial gene expression profile, altering mitochondrial structure and decreasing mitochondrial function [58]. This result suggests that mitochondrial dependence on YY1 is different in activated muscle stem cells versus differentiated muscle cells. On the other hand, YY1-mediated stabilization of the HIF1α protein was lost in activated Pax7^+^ cells lacking the YY1 gene, provoking the loss of the expression of glycolytic HIF1-responsive genes [57]. Overall, YY1 plays a vital role during satellite cell activation by favoring the HIF1a-mediated expression program of glycolytic genes and repressing mitochondrial function.

### YY1 Regulates Hematopoietic Stem Cells

Both self-renewal and differentiation potentials enable hematopoietic stem cells (HSCs) to maintain the entire hematopoietic system, which was clearly shown in bone marrow transplantation experiments performed in lethally irradiated mice. Specifically, the LSK (Lin^−^Sca^hi^c-kit^hi^ cells) compartment was shown to contain cells with the ability to repopulate the bone marrow. Pan X et al. demonstrated that mice undergoing transplantation of bone marrow progenitors ectopically expressing the YY1 gene increased LSK population in comparison with mice transplanted with bone marrow progenitors transduced with an empty vector [71]. Importantly, in-depth analysis of LSK population demonstrated that ectopic YY1 expression increased the LT-HSC population defined as CD48^−^CD150^+^ cells, highlighting that YY1 enhances the self-renewal potential of these cells [71]. Similarly, the levels of short-term (ST)-HSCs, multipotent progenitors, myeloid progenitors, monocytes, and neutrophils were also increased 30 weeks after bone marrow transplantation, suggesting that YY1 mediates the multipotency and differentiation potential of HSCs [54]. Additionally, YY1 ablation decreased these populations (Table 1). Thus, YY1 seems to regulate the stemness potential of HSCs.

To further assess the relationship between YY1 and self-renewal capacity, competitive congenic bone marrow transplantations were performed in lethally irradiated CD45.1^+^ mice by using a mixture of bone marrow progenitors from the donors harboring the CD45.1^+^ wild-type genotype with those carrying either the CD45.2^+^ Mx1-Cre YY1^f/f^ or CD45.2^+^ Mx1-Cre genotypes. A very low presence of CD45.2^+^ cells lacking YY1 expression was detected in the reconstituted hematopoietic system compared to that obtained in the case of either CD45.1^+^ wild-type- or CD45.2^+^ Mx1-Cre-transplanted cells. Additionally, no CD45.2^+^ cells with absent YY1 expression were found in the secondary bone marrow transplantations [54]. This result was explained by the fact that YY1 ablation exhausted the HSC population by inducing cell cycle progression and compromising the quiescence state of the cells. On the other hand, the REPO domain of YY1 can recruit the PRC2 complex to DNA [2]; thus, Zhanping and colleagues analyzed whether the REPO domain is implicated in the pro-stemness effects of YY1 on HSCs. Ectopic expression of a YY1 variant lacking the REPO domain rescued the loss of the YY1 phenotype, similar to wild-type YY1, suggesting that YY1-mediated PRC2 recruitment to DNA is dispensable for the regulation of the stemness potential of HSCs. Similarly, the YY1-PRC2 axis was not overrepresented in gene regulation in ESCs [48]. Overall, YY1 plays a crucial role in the hematopoietic system by regulating HSC stemness.

Neither YY1 ablation nor ectopic expression was associated with the emergence of hematological malignancies; however, YY1 overexpression increased the myeloid lineage [54, 71]. Accumulated mutations [72, 73] and epigenetic changes [74] in the HSC population have been associated with some types of myeloid leukemia; thus, detection of high expression levels of YY1 in some patients with myeloid leukemia cancers suggests that YY1 may play an oncogenic role [75]. Hence, it has been demonstrated that YY1 is associated with antitumor therapy resistance of leukemia cells [76].

## YY1 Regulates Stem Cells Derived from the Endoderm Lineage

The endoderm is the innermost gem layer that generates digestive and respiratory tubes. The role of YY1 in the development of the intestine and lung was investigated by using conditional mouse models (Table 1).

### YY1 Regulates Gut Development

The association of YY1 with the stemness potential is evident in the gut development. Specifically, YY1 knockout in the intestinal epithelium inhibited villus growth during intestinal development, and maximal reduction of the growth was observed at E18.5 [59]. This effect was attributed to the deficient differentiation potential of enterocytes. Notably, the loss of YY1 in the intestinal epithelium inhibited gene expression associated with mitochondrial function [59]. Even though YY1 expression was ablated as early as E9.5, the YY1 ablation phenotype was discernible until E16.5, during which higher expression of mitochondria-associated genes is needed for normal development of the intestinal epithelium [59]. This finding suggested that YY1 regulates later stages of intestinal development by mediating the expression of mitochondria-associated genes. Similarly, enterocyte differentiation in the later stages of intestinal development was decreased after the deletion of the mitochondrial transcription factor TFAM [77]. Alterations in the relationship between YY1 and mitochondrial function in the intestine have clinical implications. Necrotizing enterocolitis (NEC) is a pathologic human condition characterized by gut development delay and is fatal in preterm infants. The samples from patients diagnosed with NEC showed downregulation of direct targets of YY1 associated with mitochondrial function [59]. Thus, YY1 regulates the differentiation potential of enterocytes during intestinal development.

### YY1 Regulates Adult Intestinal Stem Cells

The adult intestine is another organ with high cell turnover rates, suggesting the presence of a cell population with stemness potential. Specifically, Lgr5^+^ intestinal stem cells generate all cell types of the gut epithelium. Perekatt and colleagues ablated YY1 expression in Lgr5^+^ intestinal stem cells and tracked the cells in an *in vivo* model to demonstrate the regulatory role of YY1. The results indicated that Lgr5^+^ intestinal stem cells lacking YY1 expression abandoned their crypt-base localization and migrated onto the villi to undergo differentiation and cell death [60]. The vacant stem niche was filled with intestinal stem cells expressing YY1 [60]. It was performed gene expression microarrays and YY1 ChIP-seq analyses of crypt epithelial cells lacking YY1 expression to clarify the molecular mechanisms mediated by YY1 in these cells. The loss of YY1 inhibited the expression of the genes encoding the mitochondrial complex I components and transactivated genes associated with cell cycle progression and RNA processing [60]. Consistently, the stemness potential of adult intestinal stem cells was compromised due to mitochondrial dysfunction resulting from the loss of HSP60, a mitochondrial chaperone [78], or ablation of the mitochondrial transcription factor TFAM [77]. Interestingly, *in vitro* assays showed that YY1 downregulation compromised *in vitro* organoid generation due to ROS inhibition, indicating that mitochondrial function is required for the stem cell phenotype [60]. Thus, YY1 maintains adult intestinal stemness by inducing a transcriptional program needed for mitochondrial function and cellular metabolism.

### Role of YY1 in Lung Development

As previously mentioned, ablation of the YY1 gene results in peri-implantation lethality. To circumvent this, the animals harboring YY1 hypomorphic alleles (YY1^flox − neo/flox−neo^) were crossed with mice heterozygous for the YY1 null allele (YY1^−/+^). Thus, generated mouse line was genotyped as YY1^flox − neo/−^ and displayed YY1 expression levels equal to 25 % of the normal expression. Approximately 50 % of the embryos harboring this genotype were alive at birth; however, the animals succumbed within two days postnatally due to inability to breathe attributed to the collapse of alveoli [79]. In agreement with this finding, Bérube and colleagues showed that conditional ablation of the YY1 gene in the lung mesenchyme induced structural alterations in embryonic lungs at E18.5, such as a decrease in club and pneumocyte type I cells. [61]. Interestingly, ablation of YY1 in the lung epithelium in another conditional mouse model also induced respiratory complications at birth that ended with postnatal death. Structural analyses of organogenesis of embryonic lung in these mice showed that YY1 regulated apoptosis and proliferation and is thus essential for lung branching [62]. Moreover, YY1 ablation diminished the density and diameter of the ring cartilage distributed along the trachea [62]. YY1 ablation in the pulmonary epithelium did not alter the precursors of basal cells expressing p63; however, this manipulation decreased the number of ciliated, club, and goblet cells, suggesting compromised differentiation potential [62]. Overall, lung development of various conditional KO mouse models targeting the YY1 gene demonstrated the regulatory role of YY1 in the differentiation potential of committed pulmonary progenitor cells.

## Role of YY1 in the Cancer Stem Cell Phenotype

Previous sections described the role of YY1 in the regulation of the stemness potential of embryonic stem, lineage-committed progenitor, and adult stem cells (Table 1). Interestingly, in certain biological settings, YY1 modulates stemness potential by regulating the transcriptional landscape associated with mitochondrial function [50, 59, 60, 69]. In cancer, the CSC phenotype has been associated with mitochondrial function, and targeting this function is emerging as a potential anticancer therapy for specific elimination of CSCs [80, 81]. Pancreatic adenocarcinoma stem cells are sensitive to metformin treatment that inhibits mitochondrial function due to a decrease in the expression of PCG-1α, a mitochondrial transcription factor [82]. PCG-1α interacts with YY1, inducing co-binding to the promoter sequences of mitochondrial genes and activating their transcription [83]. Similarly, ectopic downregulation of either YY1 or PCG-1α compromises mitochondrial function, reducing the oxygen consumption rate and ATP production levels [83, 84]. Importantly, mTOR promoted physical interaction between YY1 and PCG-1α enhancing mitochondrial function [83]. mTOR inhibition compromised CSC traits [85, 86] by modulating mitochondrial function in several types of cancers, such as pancreatic, ovarian, and bone cancers [82, 87–89], suggesting that the YY1-mTOR-PGC-1α axis plays the key role in the biology of CSCs.

YY1 is implicated in the regulation of the Core regulatory circuitry in ESCs. Analysis of the data retrieved from an experimental protein database of cancer patient tissues enabled stratification of 17 types of cancer based on the expression patterns of YY1 and stem cell transcription factors. The expression levels of Sox2 and Oct4 were significantly associated with each other in all tested cancer types [90]. Notably, YY1 and Oct4 protein levels strongly correlated with each other in all analyzed cancer types [90]. Additionally, high expression levels of both YY1 and Sox2 were common in specific types of cancer. These findings are in agreement with the fact that YY1 mediates gene expression and stabilizes the levels of proteins encoded by the Oct-4 and Sox-2 genes [41]. Since Sox2 and Oct4 are essential for ESC biology [91]; codetection of these proteins enables experimental isolation of CSCs [92, 93], and targeting of these proteins inhibits tumor growth [94]; thus, associations of YY1 with Oct4 and Sox2 suggest a possible role for YY1 in the CSC phenotype.

### Role of YY1 in Brain CSCs

**Fig. 1:**
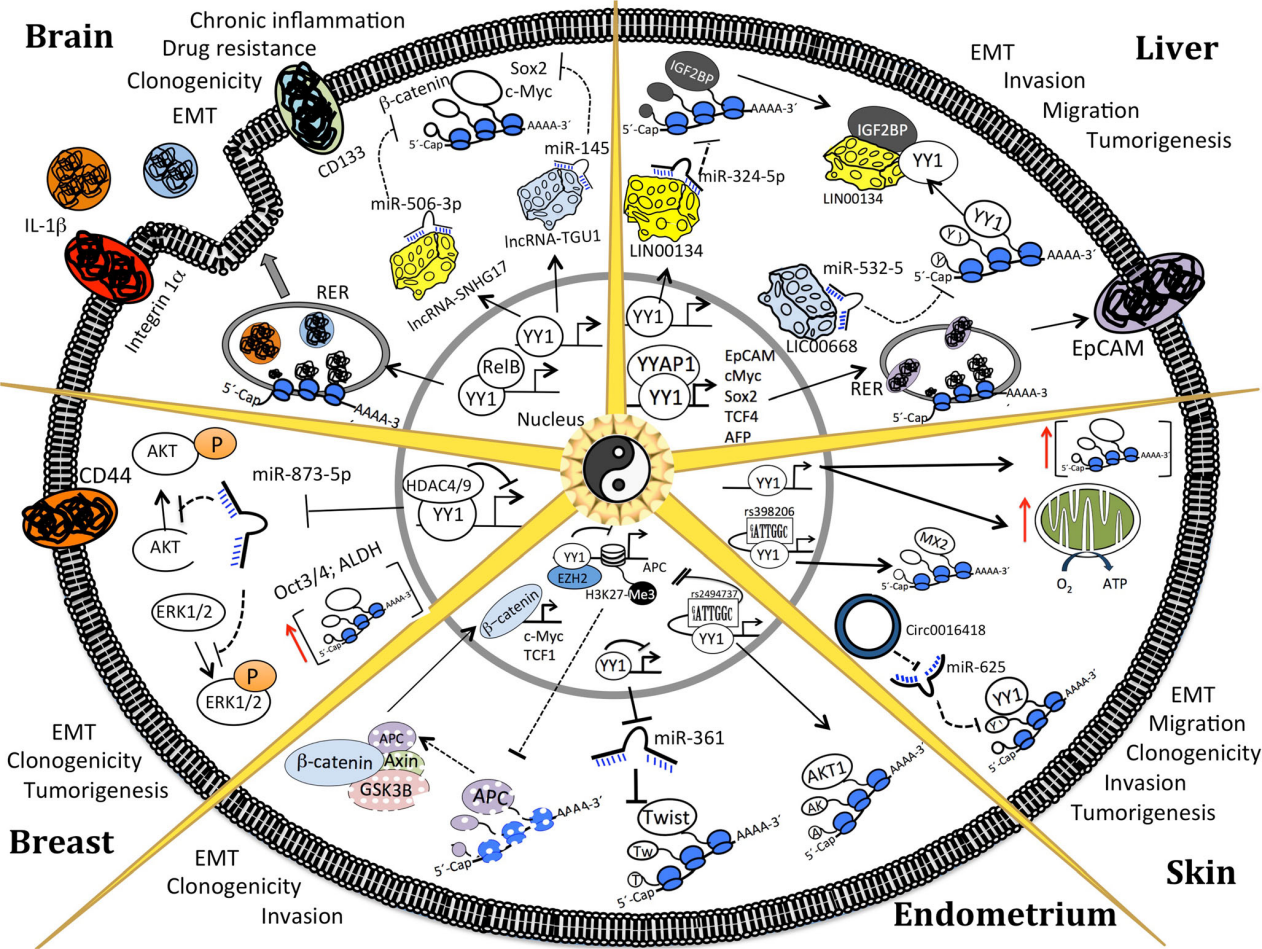
Molecular pathways modulated by YY1 in CSCs. Molecular pathways altered by YY1 in CSCs from endometrial, brain, liver, skin, and breast cancers are shown. RER, rough endoplasmic reticulum; EMT, epithelial-mesenchymal transition

Glioma tumors are characterized by extensive infiltration of immune cells, contributing to the formation of a tumor microenvironment that triggers metastasis and resistance to anticancer treatments [95]. On the other hand, specific signaling pathways are associated with the CSC phenotype, such as NFκB, Wnt, and Notch [95]. RelB is a member of the NFκB signaling pathway and generates a specific cytokine profile responsible for myeloid cell recruitment to glioblastoma tumors (Fig. 1). Importantly, YY1 cointeracts with RelB in the promoter sequences of these cytokine genes to govern their transcriptional activation [96]. Thus, YY1 might support the CSC phenotype by contributing to the generation of an immunosuppressive microenvironment. The Wnt/CTNNB1 pathway is well known to be associated with both tumorigenesis and CSC phenotype [97, 98]. YY1 upregulates the expression lncRNA-SNHG17, which extends the half-life of CTNNB1 by sponging its direct negative regulator, miR-506-3p [99]. Significantly, the downregulation of lncRNA-SNHG17 inhibits tumor growth *in vitro* and *in vivo* [99]. Thus, YY1 regulates the activation of the Wnt1 pathway in glioma cells. The Notch pathway is another signaling pathway implicated in the CSC phenotype. lncRNA taurine upregulated gene 1 (TUG1) is a direct target gene of Notch that maintains the glioma CSC phenotype manifested as high expression of CD15, Sox2, and Myc genes and an increase in neurosphere formation [23]. Significantly, the pro-stem functions of TUG1 are mediated by sponging miRNA-145 to impede the miRNA-145-dependent degradation of Sox2 and Myc mRNAs [23]. Moreover, undifferentiated state of the cells is maintained by TUG1 through cointeraction with the YY1-PRC2 complex that induces silencing of differentiation-associated genes, such as BDNF, NGF, and NTF3 [23]. Therefore, YY1 appears to be an essential branch of several signaling pathways associated with the CSC phenotype in brain tumors (Fig. 1).

Glioblastoma multiforme (GBM) is one of the most aggressive brain malignancies and is characterized by the lowest patient survival rate due to chemotherapy resistance [100]. YY1 appears to govern this outcome by enhancing the properties of CSCs. Specifically, YY1 was overexpressed in cisplatin-resistant glioblastoma cells. YY1 loss-of-function assays demonstrated that YY1 mediates the growth of tumor spheres and expression of CSC markers, such as CD133, STAT3, and integrin-α6 [101]. Importantly, the downregulation of YY1 by miR-186 was sufficient to induce cisplatin-mediated cell death [101]. On the other hand, temozolomide is used as first-line chemotherapy in newly diagnosed glioblastoma patients and in patients with refractory anaplastic astrocytomas [102]. shRNA-mediated downregulation of YY1 in temozolomide-resistant glioblastoma multiforme cells was shown to compromise the CSC population evaluated based on CD133 expression and spheroid and colony formation assays [103]. Notably, miR-7-5p is a negative regulator of YY1, and the levels of miR-7-5p expression were downregulated in temozolomide-resistant glioblastoma cells; thus, miR-7-5p overexpression reversed temozolomide resistance *in vivo* [103]. Overall, YY1 contributes to anticancer therapy resistance in GBM by enhancing the CSC phenotype (Fig. 1).

### Role of YY1 in Hepatic CSCs

Hepatocellular carcinoma (HCC) is one of the most aggressive tumors. The levels of YY1 expression were shown to be higher in the samples of patient with HCC than that in the normal counterpart tissue samples [9, 10, 104]. Moreover, YY1 has been shown to trigger molecular mechanisms leading to HCC carcinogenesis [105] and the development of worse clinical phenotypes, such as angiogenesis [27] and antineoplastic drug resistance [104]. YY1 enhanced the migration, invasion, and epithelial-mesenchymal transition of HCCs by inducing the expression of linc01134, a lncRNA expressed at a high level in HCC [9]. linc01134 impeded miRNA-324-5p-mediated downregulation of IGF2BP1 by sponging miRNA-324-5p [9]. IGF2BP1 interacts with and extends the half-life of YY1 mRNA; thus, YY1 can regulate its own expression by regulating linc01134 expression in HCC [9]. Importantly, ectopic YY1 expression was sufficient to trigger epithelial-mesenchymal transition and invasive and migratory potential of the cells [9]. Therefore, YY1 triggers aggressive phenotypes in HCC in a linc01134-dependent and linc01134-independent manner. Another study demonstrated that the levels of YY1 expression were protected by linC00668, which sponges miR-532-5 to inhibit miR-532-5-mediated degradation of YY1 [10]. Importantly, both YY1 and linC00668 enhanced the proliferation, EMT, migration, and invasiveness of the cells [10]. Interestingly, ectopic YY1 expression rescued the inhibition of tumor growth *in vivo* and *in vitro* induced by downregulation of linc00668 [10]. Therefore, YY1 is an oncogenic regulator of HCC located upstream of linc00668 or linc01134. Overall, these studies showed that YY1 extensively regulates cellular processes strongly associated with the CSC phenotype, such as EMT, angiogenesis, migration, and invasion [106]. Thus, YY1 is expected to regulate the CSC phenotype in HCC.

Investigation of the molecular pathways in CSCs obtained from the samples of HCC patients provided new insight into the role of YY1 in the CSC phenotype. Analysis of transcriptomic datasets generated using the samples of HCC patients enriched in CSCs, which were selected based on the high expression levels of the EpCAM and AFP genes, which are well-known CSC biomarkers [107], indicated that the levels of the expression of direct transcriptional targets of YY1 were the highest [108]. One of these genes, YY1AP, was further characterized as a driver of CSC phenotype because the lowest survival rates of patient correlated with high copy number of YY1AP [108]. Similarly, YY1AP enhanced the *in vitro* and *in vivo* tumorigenic potential in YY1AP loss-of-function assays [108]. Significantly, YY1AP requires an association with transcriptional activation of the expression of the EpCAM gene induced by YY1 [108]. Thus, YY1 regulates the YY1AP-mediated CSC phenotype in HCC.

### Role of YY1 in Endometrial CSCs

Endometrial cancer is the commonest gynecologic malignancy. Previous studies showed that the PRC2 complex containing EZH2 plays a crucial role in endometrial cancer by promoting the proliferation, migration, and metastasis [109, 110]. These effects are partially attributed to EZH2-mediated silencing of tumor suppressor miRNAs [111, 112]. miR-101 downregulates EZH2 and was thus overexpressed to investigate new tumor suppressor miRNAs potentially repressed by EZH2 [113]. miRNA-361 was identified out of miRNAs upregulated by miR-101 overexpression due to higher expression in normal endometrial tissues than that in their cancerous counterpart tissues [113]. The results of the miRNA-361 gain- and loss-of-function assays indicated that tumor suppressor functions miRNA-361 involve inhibition of invasiveness, spheroid formation potential, clonogenicity, EMT, and expression of CSC biomarkers [113]. YY1 is required to promote the recruitment of EZH2 to the miRNA-361 promoter [113], suggesting that YY1 governs the CSC phenotype in endometrial cancer. Additionally, YY1 was shown to silence APC gene expression in endometrial cancer cells by enhancing EZH2-mediated H3K27me3 deposition on the APC promoter [114]. YY1-mediated APC silencing relieved β-catenin repression, allowing the transactivation of its direct gene targets that enhanced tumorigenic potential both *in vitro* and *in vivo* [114]. On the other hand, YY1 regulates the oncogenic PI3K/AKT signaling pathway in endometrial tumors. The results of genome-wide association studies indicated that the minor allele of the SNP rs2494737 located at the 14q32.33 locus was associated with increased risk for endometrial tumors [115]. The results of ChIP-3 C, gene reporter, EMSA, and YY1 loss-of-function assays demonstrated that this allele generates a YY1-binding site in a distal regulatory region of the AKT1 gene that enhanced transcriptional activation [115]. Both the PI3K/AKT and Wnt/b-catenin signaling pathways modulate the CSC phenotype in endometrial cancer [116]; thus, YY1 might regulate the CSC phenotype, leading to worse clinical outcomes. Evidence supporting this hypothesis includes YY1 overexpression in tumor samples of the patients and inhibition of *in vitro* and *in vivo* tumorigenic potential by YY1 downregulation [114].

### Role of YY1 in Skin CSCs

Melanoma is one of the deadliest skin cancers worldwide [117]. Apparently, YY1 plays a key role in melanoma because its expression levels correlate with advanced tumor stages [26, 118] and ectopic YY1 downregulation inhibits the proliferation, migration, invasion, and resistance to anticancer therapies [26, 118, 119]. Interestingly, the rs398206-A allele is associated with increased risk for melanoma because it generates to a YY1-binding site that results in transcriptional activation of the MX2 gene, which enhances melanomagenesis on a BRAF^V600E^ background [120]. YY1 is essential for the generation of embryonic melanocytic lineage and adult melanocyte stem cells [53], and melanoma is strongly associated with the CSC phenotype [121–124]; thus, YY1 could be regulating the melanoma CSC phenotype.

The results of the experiments in a mouse melanoma model expressing human oncogene N-Ras^Q61K^ only in melanocytic lineage on an INK4a-deficient background [125] demonstrated that YY1 is coexpressed with a melanocytic marker in both hair follicles and skin melanomas [50]. This finding suggests a possible association between YY1 and undifferentiated cellular state. Tyr::CreER^T2^;YY1^fl/fl^ or Tyr::CreER^T2^;YY1^fl/wt^ inducible floxed YY1 mice were crossed with the model melanoma strain mentioned above to generate a mouse line with inducible YY1 ablation in melanoma cells aiming to investigate the role of YY1 in melanoma. Ablation of only one YY1 allele was sufficient to inhibit melanoma formation, indicating that melanogenesis is linked to YY1 expression [50]. RNA-seq and YY1 ChIP-Seq analyses were performed after RNAi-mediated knockdown of YY1 to extend these observations to human melanoma cells [50]. siRNA-mediated YY1 downregulation decreased the expression of direct YY1 target genes associated with the mitochondrial electron transfer chain, tricarboxylic acid cycle, protein synthesis, and other fundamental metabolic pathways [50]. Similarly, functional assays demonstrated that mitochondrial bioenergetics, protein synthesis, and other metabolic pathways were compromised by the loss of YY1 expression in melanoma cells [50]. Thus, YY1 apparently regulates CSC metabolism in melanoma (Fig. 1).

### Role of YY1 in Breast CSCs

The CSC phenotype has been extensively studied in breast cancer [126, 127], showing its association with worse clinical scenarios, such as resistance to anticancer therapies, metastasis, angiogenesis, and cancer recurrence [128, 129]. Importantly, YY1 expression levels correlate with the presence of breast cancer [130, 131]. The results of YY1 loss- and gain-of-function assays showed that YY1 fuels the migratory, invasive, colony-forming, and *in vivo* tumorigenic potentials of breast cancer cells [130]. Similarly, YY1 is associated with life-threatening processes, such as the development of metastasis [130, 132, 133]. Since these results are indicators of the presence of the CSC population, YY1 may play a role in the regulation of the CSC phenotype. Interestingly, YY1 decreased p27 protein levels in *in vitro* and *in vivo* assays by enhancing Sk2-mediated p27 polyubiquitination [130]. p27 induces cell cycle inhibition by targeting the cyclin E-CDK2 complex, suggesting that YY1 contributes to oncogenic behavior by enabling cell cycle progression [134]. Interestingly, a decrease or lack of p27 expression have been associated with the stemness potential of human ESCs [135] and the generation of induced pluripotent stem cells [136], suggesting that Sk2-mediated p27 degradation induced by YY1 may favor the CSC phenotype.

Another study demonstrated that breast CSCs enriched by mammosphere culture had lower NMI expression levels than that in non-CSCs [137]. The results of the NMI loss- and gain-of-function assays demonstrated that NMI decreases several CSC features, such as the expression of stem cell transcription factors, frequency of the CD44^+^CD24^−^ cell population, mammosphere-forming potential, EMT, and xenograft tumor formation [137]. These effects were attributed to NMI-mediated downregulation of the hTERT gene, a well-known pro-stemness factor in breast cancer [138, 139]. NMI lacks a DNA-interacting domain and modulates gene expression by functioning as a coactivator. NMI immunoprecipitation coupled with mass spectrometry was performed to identify transcription factors associated with NMI-mediated downregulation of hTERT; the results identified YY1 as an NMI-interacting protein. The YY1-NMI complex was able to silence the expression of the hTERT gene by binding to the hTERT promoter; however, this axis was not directly assessed in the CSC population [137]. Thus, the role of YY1 in breast CSCs was incompletely determined. Importantly, breast tumor samples were recently shown to have high levels of expression of YY1 and stem cell transcription factors, such as Oct4, Sox2, and Nanog [140]. Importantly, the ectopic modulation of YY1 demonstrated its positive regulatory role in the CSC phenotype due to an increase in several stemness traits, including the expression of stem cell transcription factors, sphere-forming potential, percentage of CD44^+^CD24^−^ cells, and the ability to form tumors *in vivo* [140]. Additionally, YY1 positively regulated the ERK1/2 and PI3K/AKT pathways, and inhibition of these pathways by small molecules decreased the tumorigenic effects of YY1 [140]. Importantly, YY1 inhibited miR879-5p expression by interacting with its promoter sequence [140]. Since miR879-5p inhibits the YY1-mediated CSC phenotype, the repressive role of YY1 in the regulation of the miR879-5p gene is required for the maintenance of the breast CSC phenotype (Fig. 1).

### Potential Role of YY1 in the CSC Niche

Microenvironment (ME) of the CSC niche provides correct signals to the CSC population that secure their stemness properties. This ME is sculpted by corrupted tumor-associated cells (TACs), such as fibroblasts, mesenchymal stem cells, immune cells, and adipocytes, that exchange certain molecules and establish specific interactions with CSCs [141]. Co-engraftment of CSCs with tumor-associated fibroblasts, which are one of the main populations mediating the formation of the CSC niche, enhances tumor growth [142], antitumor therapy resistance [143], invasion, and metastasis [144]. Targeting TACs or molecules secreted by TACs inhibits the CSC phenotype, and TACs are thus emerging as attractive targets for the development of anti-CSC therapies. Therefore, investigation of YY1-mediated functions in TACs that support the CSC niche is required to advance our understanding of the role of YY1 in the CSC phenotype. Some interesting results have been reported; however, the data on these relationships are lacking. Tumor-associated macrophages (TAMs) are M2-polarized macrophages that enhance tumor growth via their immunosuppressive functions. Macrophages expressing miR-125a were shown to suppress the polarization of the M2 phenotype to inhibit tumor growth *in vivo* [145]. Importantly, YY1 apparently promoted the development of TAMs by repressing miR-125a expression. Specifically, YY1 is able to interact with a distal regulatory element that governs miR-125a silencing in an RYBP-dependent manner [145]. Thus, the role of YY1 in various TACs supporting the CSC niche requires further investigation.

**Fig. 2:**
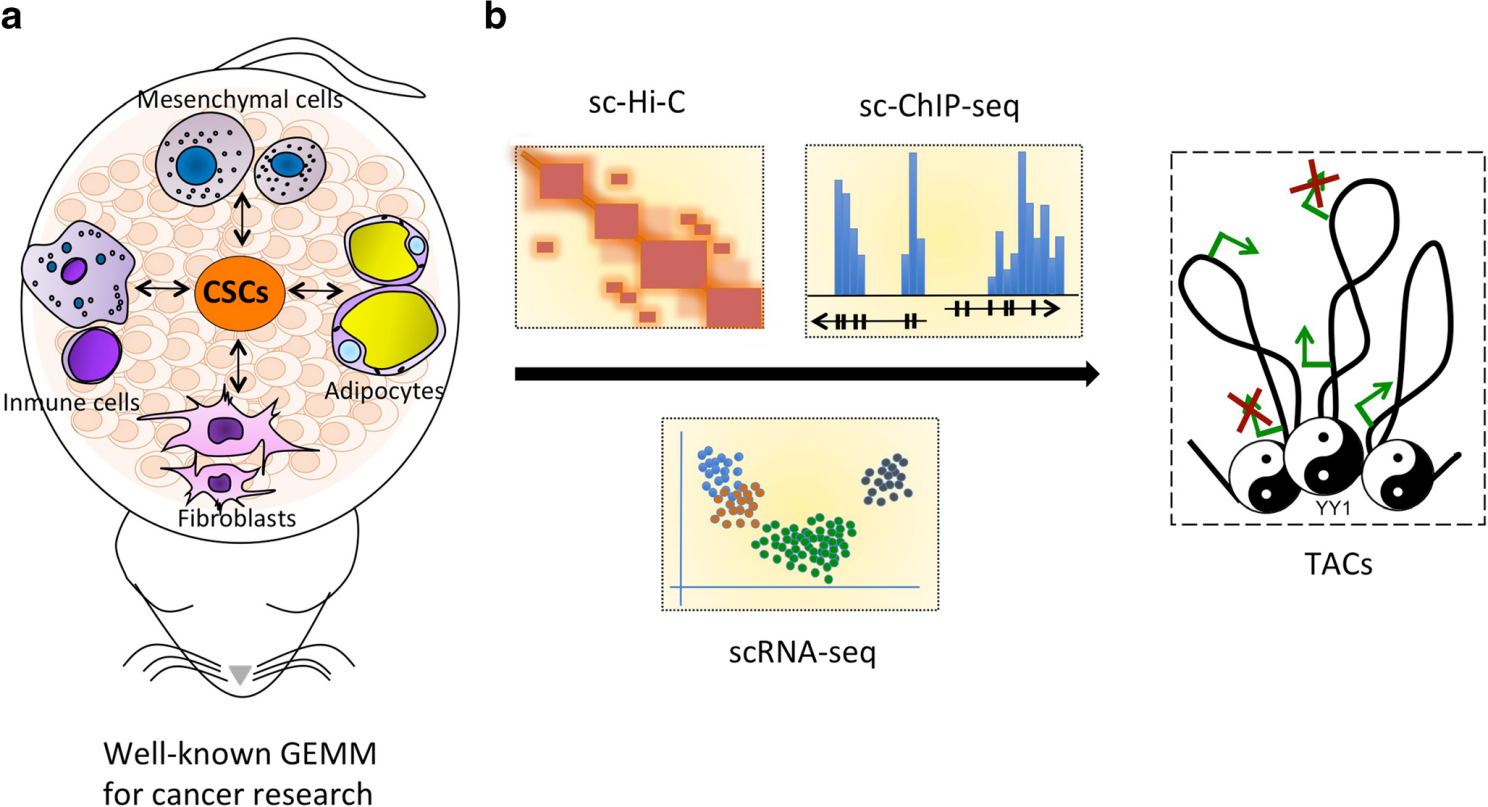
Potential role of YY1 in the regulation of the CSC niche. **a** The use of well-known genetically engineered mouse models for cancer research with conditional ablation of YY1 in specific TACs, such as mesenchymal stem cells (MSCs), immune cells, tumor-associated fibroblasts, and adipocytes, may enable to determine the role of YY1 in tumor-associated cells (TACs) regulating the CSC niche. **b** YY1 is currently conceptualized as a 3D chromatin regulator; thus, it will be of interest to determine whether YY1 imposes specific gene expression in TACs supporting the CSC niche by modulating their chromatin configuration

The lack of observations on the role of YY1 in the CSC niche may be a consequence of the lack of *in vivo* models for studies of the CSC niche. CSCs are functionally defined as the cells that can generate tumors in the limiting dilution and serial tumor transplantation assays in immunocompromised mice. Potentially, the failure of a putative CSC population to display tumorigenic behavior assessed in these *in vivo* assays indirectly reflects the addiction of CSCs to a correct CSC niche. Thus, generation of the *in vivo* models, in which TACs are assessed and modulated, such as immune cells, is required to study CSCs to gain new insight into human cancer biology. Therefore, the development of genetically engineered mice with conditional and active YY1 ablation in specific TACs is needed to circumvent this limitation. Since YY1 mediates specific 3D chromatin configurations needed for cell identity, we suggest that YY1 may govern both CSCs and the CSC niche by imposing cell type-specific chromatin configurations. Thus, single-cell high-throughput technologies, such as single-cell ChIP-seq [146, 147] and single-cell chromatin conformation capture [148, 149], may be able to promote this area of research (Fig. 2).

## Concluding Remarks

The use of genetically engineered mouse lines coupled with high-throughput technologies of molecular biology enabled to investigate the role of YY1 in the stemness potential in limited scenarios during embryonic development and in some adult stem cells. Thus, YY1 governs the stemness potential by regulating the transcriptional landscape associated with cellular metabolism, energetics, and cell death. In cancer, in addition to the modulation of molecular processes similar to those in normal stem cells, YY1 governs the CSC phenotype via tumor-dependent molecular events (Fig. 1). This effect may be a consequence of the presence of various tissue-specific selection pressures. On the other hand, analysis of the regulatory effects of YY1 on TACs supporting the CSC niche, which is an essentially unexplored area, is needed to enrich our understanding of the role of YY1 in the CSC phenotype. Notably, most YY1-mediated actions regulating the CSC phenotype have been polarized to YY1-mediated transcriptional regulation of the target genes. However, YY1 is emerging as a dynamic regulator of the three-dimensional chromatin configuration. Since 3D genome organization dictates cell identity and its dynamic rearrangement is associated with cellular responsiveness [150, 151], we envision that YY1 may induce specific chromatin conformation states needed for TACs that support the CSC niche. Thus, *in vitro* and *in vivo* models for the investigations of the role of YY1 in the chromatin configuration in specific TACs, which support the CSC niche, may be able to identify new druggable molecular targets.

## Data Availability

All data presented in this review are totally available and present in the text.

## Abbreviations

YY1: yin yang-1
CSCs: cancer stem cells
TACs: tumor-associated cells
PRC2: polycomb repressive complex 2
lncRNA: long non-coding RNA
SC: stem cell
ICM: inner cell mass
EMT: epithelial-mesenchymal transition
ESC: embryonic stem cell
NPC: neural progenitor cell
ChIP: chromatin immunoprecipitation
CPC: cardiac progenitor cell
MyHC: myosin heacy chain
NEC: necrotizing enterocolitis
HSC: hematopoietic stem cell
PGC-1α: peroxisome proliferator-activated receptor gamma coactivator 1-alpha
NFKB: nuclear factor kappa B
ME: microenvironment
